# Oil type and cross‐linking influence growth of *Aureobasidium melanogenum* on vegetable oils as a single carbon source

**DOI:** 10.1002/mbo3.605

**Published:** 2018-03-12

**Authors:** Loes H. M. Peeters, Hendrik P. Huinink, Benjamin Voogt, Olaf C. G. Adan

**Affiliations:** ^1^ Department of Applied Physics Eindhoven University of Technology Eindhoven The Netherlands

**Keywords:** *Aureobasidium melanogenum*, biofilm biology, cross‐linking, metabolic activity, oil conversion, vegetable oils

## Abstract

*Aureobasidium melanogenum* is the main fungus found in a spontaneously formed biofilm on a oil‐treated wood. This dark colored biofilm functions as a protective coating. To better understand biofilm formation, in this study *A. melanogenum* was cultured on olive oil and raw linseed oil. Metabolic activity and oil conversion were measured. The results show that *A. melanogenum* is able to grow on linseed oil and olive oil as a single carbon source. The fungus produces the enzyme lipase to convert the oil into fatty acids and glycerol. Metabolic activity and oil conversion were equal on linseed oil and olive oil. The fungus was not able to grow on severe cross‐linked linseed oil, meaning that the degree of cross‐linking of the oil is important for growth of *A. melanogenum*. Dark coloring of the colony was seen on linseed oil, which might be a stress response on the presence of autoxidation products in linseed oil. The colony on olive oil showed delayed melanin production indicating an inhibitory effect of olive oil on melanin production.

## 1 INTRODUCTION

Normally, fungal growth on wood and coatings is unwanted and associated with wood degradation (Bardage, 1998; Gobakken et al., 2010; Harvath et al., 1976). Coloring due to fungal growth is often not appreciated. However, a uniform dark‐colored biofilm containing *Aureobasidium melanogenum* can be used as a protective wood coating (Sailer et al., 2010; Van Nieuwenhuijzen et al., 2016; Van Nieuwenhuijzen et al., 2015). When wood is impregnated with vegetable oils and placed outdoors, this biofilm develops on the wood surface. Such a biofilm has several advantages: the system is environmentally friendly, no harmful chemicals are involved in the procedure, it has self‐healing properties, and low maintenance costs. A major disadvantage of spontaneous biofilm formation in an outdoor situation is that it takes 1–3 years to form a uniform colored layer. For large‐scale application, a method to accelerate biofilm formation is needed which requires better understanding of the biofilm formation process and the safety issues regarding the use of microorganisms and the possible health effects. This manuscript focuses on growth of *A. melanogenum* on oil in order to better understand the biofilm formation process.

*Aureobasidium melanogenum* was formerly known as *Aureobasidium pullulans*. In 2008, this fungus was redefined as a variety of *A. pullulans*, namely *Aureobasidium pullulans* var. *melanogenum* (Zalar et al., 2008). In 2014, this variety was defined as a separate species named *A. melanogenum* (Gostinčar et al., 2014). *Aureobasidium melanogenum* is a pleomorphic fungus that shows phenotypic plasticity, meaning that the fungus grows in different shapes and is able to change morphology, physiological state, and/or behavior (Slepecky & Starmer, 2009; Van Nieuwenhuijzen et al., 2016). *Aureobasidium melanogenum* has both a yeast‐like and a hyphal growth mode. Furthermore, it can form chlamydospores—big cells with a typical dark color due to production of melanin (Kocková‐Kratochvílová et al., 1980; Zalar et al., 2008). *Aureobasidium melanogenum* is found on many different surfaces, for instance, on leafs (McGrath & Andrews, 2007), wood, painted material (Harvath et al., 1976; Shirakawa et al., 2002), and rocks (Urzì et al., 1999). Recently, it was found that *A. melanogenum* is the main fungus in dark‐colored biofilms on oil‐impregnated wood (Van Nieuwenhuijzen et al., 2016).

Biofilm formation was further investigated by Van Nieuwenhuijzen et al. (2015). They studied the influence of wood species, oil type, and climate conditions on the formation of a uniform biofilm. An assessment method based on visual stain coverage and quantification of darkness was developed to detect biofilms on wood. This method demonstrated reproducible biofilm formation on pine sapwood treated with raw linseed oil. Furthermore, when using olive oil, biofilm formation was demonstrated on all wood types, while treatment with stand linseed oil (polymerized linseed oil) did not lead to biofilm formation. Van Nieuwenhuijzen concluded that oil is essential for biofilm formation and that the oil type is more important to biofilm formation than the wood type.

The exact influence of oil type on biofilm formation is not clear. The molecular mobility of the oil seems to influence the availability of food for the fungus, as seen for stand oil, where no biofilm formation was observed (Van Nieuwenhuijzen et al., 2015). An important property of oil, which effects both nutritive value and physical properties like mobility, is the fatty acid composition. Fatty acids can be saturated or unsaturated. Saturated fatty acids do not have double bonds in their carbon chain. Unsaturated fatty acids contain one or more double bonds and make the oil more mobile (Berg, Tymoczko, & Stryer, 2002). Polyunsaturated fatty acids contain two or more double bonds and tend to cross‐link with each other in the presence of oxygen. The more double bonds, the more fatty acids tend to cross‐link. Polyunsaturated fatty acids are therefore less stable and when cross‐linking occurs, the oil becomes less mobile. Moreover, fatty acids with one carbon atom between two double bonds are susceptible to cross‐linking, since the hydrogen atoms connected to this carbon atom are likely to be dissociated by oxygen (Porter et al., 1995).

It is not clear which component of the oil‐treated wood is used as a nutrient by *A. melanogenum* in the biofilm. Possible carbon sources include oil, components from the wood itself, or dust and pollen that stick to the wood surface. Also, other microorganisms (precursors) can colonize the wood, enabling growth of *A. melanogenum*. It seems likely that *A. melanogenum* can use oil as a nutritient since it is known to produce lipase, an enzyme that hydrolyzes oil molecules (triglycerides) into the potential carbon sources glycerol and fatty acids (Chi et al., 2009; Leathers et al., 2013; Leelaruji et al., 2013; Liu et al., 2008; Wang et al., 2007; Wongwatanapaiboon et al., 2016). Moreover, *Aureobasidium* is known to grow on paints containing linseed oil (Bardage, 1998; Harvath et al., 1976). Until now, however, little is known of the mechanism of oil consumption by *A. melanogenum*. The effect of the fatty acid composition, degree of cross‐linking, and mobility of the oil on growth of *A. melanogenum* is not clear. For large‐scale industrial production of the biofilm, understanding of the growth mechanisms of *A. melanogenum* on oil‐treated wood is crucial.

In this study, growth of *A. melanogenum* on oil as a single carbon source was investigated. First, the effect of olive and raw linseed oil was studied by comparing growth on these oils. Second, the mechanism of oil consumption of these two oils by *A. melanogenum* and the role of lipase was studied. Fungal growth was followed visually by taking macroscopic and microscopic pictures of the samples and relating them to metabolic activity. Hydrolysis of oil into fatty acids and glycerol was monitored with Fourier transform infrared spectroscopy (FT‐IR). Nuclear magnetic resonance (NMR) imaging was used to study changes in the mobility of the oil over time.

## 2 MATERIALS AND METHODS

### 2.1 Preparation of cell suspensions

Malt extract agar (MEA, Oxoid) plates were inoculated with *A. melanogenum* (strain CBS 140241 from Westerdijk Fungal Biodiversity Center, Utrecht, The Netherlands) and were cultured at 23°C for 7–14 days. Cells from the boundary of the colony were removed and diluted in minimal medium (MM) for *Aspergillus niger* supplemented with 2% glucose (De Vries et al., 2004). The suspension was adjusted to a concentration of 0.05 × 10^6^ cells/ml and was shaken for 24 hr (200 rpm at 25°C). Subsequently, the suspension was two times pelleted by centrifugation (2,000*g*, 5 min), followed by washing with autoclaved demineralized water. Then, the suspension was pelleted, suspended in five times the initial volume of MM with 2% glucose and shaken for 24 hr (200 rpm at 25°C). Finally, the suspension was washed once and diluted in MM until a concentration of 1 × 10^7^ cells/ml. This suspension was used as inoculum.

### 2.2 Oils

Growth experiments were done on the following oils: raw linseed oil (further addressed as linseed oil, Vliegenthart B.V., Tiel, The Netherlands, acid value = 0.4 mg KOH per g, free fatty acids: 0.2 g per 100 g (Nielsen, 2013)) and olive oil (Wijnimport Van der Steen B.V., Vught, The Netherlands, acid value = 0.9 mg KOH per g, free fatty acids: 0.45 g per 100 g). The fatty acid composition of the used oils was analyzed using gas chromatography (GC) by Akzo Nobel (Table 1). Linseed oil contains mostly polyunsaturated fatty acids, and olive oil contains mostly monounsaturated fatty acids.

**Table 1:** Fatty acid composition of olive and linseed oil analyzed by GC analysis after saponification of the oils with tetramethylammoniumhydroxide in methanol and methylation of the fatty acids with methyliodide in dimethylformamide (internal procedure by Akzo Nobel)

Fatty acids		Olive oil	Linseed oil
Saturated fatty acids	C16:0 Palmitic	11	6
	C18:0 Stearic	4	4
Monounsaturated fatty acids	C18:1 Oleic	80	22
Diunsaturated fatty acids	C18:2 Linoleic	5	17
Polyunsaturated fatty acids	C18:3 Linolenic	—	51

### 2.3 Growth experiments

To mimic the outdoor situation of biofilm formation on a wood surface, *A. melanogenum* was cultured on the surface of filters instead of in liquid cultures. The advantage of this system is that medium composition and stirring speed do not influence the results. A hydrophilic polyvinylidene fluoride filter (PVDF) with pore size 0.2 μm (ø 13 mm, Merck Millipore) was inoculated with 5 μl of cell suspension, dried at room conditions for at least 15 min until the fluid of the suspension has evaporated and placed on 20 μl of oil in a Petri dish.

To study the growth on cross‐linked linseed oil, linseed oil was heat‐treated until a rigid, cross‐linked layer was obtained. This treatment was done by placing a filter on 20 μl of linseed oil and incubating in a ventilated oven at 50°C for 12 days. Subsequently, this filter was inoculated with 5 μl of cell suspension and dried at room conditions for at least 90 min until the fluid of the suspension has evaporated.

**Figure 1:**
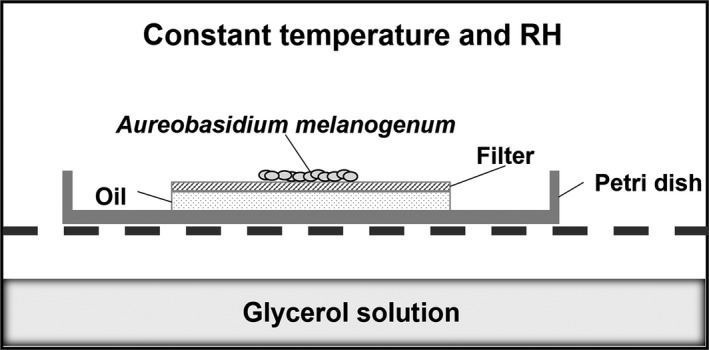
Schematic representation of the experimental setup used for growth experiments. A filter was inoculated with *Aureobasidium melanogenum* and placed on 20 μl of oil in a Petri dish. Petri dishes are incubated in a box above a 14.12% w/w glycerol solution that controls relative humidity (RH). RH during the experiments was 97%, temperature was kept at 23°C ± 1°C

All the samples were incubated at 23°C ± 1°C in a closed box with a relative humidity of 97%, controlled with aqueous glycerol solution (14.12% w/w glycerol in water) that was not in direct contact with the filter (Forney & Brandl, 1992). A scheme of the setup is shown in Figure 1.

At 3, 7, 16, 31, 64, and 106 days of incubation, pictures were taken of three samples per group. Subsequently, these samples were subjected to an MTT (3‐(4,5‐dimethylthiazol‐2‐yl)‐2,5‐diphenyltetrazolium bromide) assay for metabolic activity. At 3, 7, 11, 15, 18, 28, 31, and 64 days, three other samples were analyzed with FT‐IR to study oil conversion. An in‐depth description of the imaging, MTT analysis and FT‐IR measurements is given in the subsequent subsections.

### 2.4 Imaging

The samples were imaged with an 18.0‐megapixel digital reflex camera Canon EOS 700D and Tamron SP 60 mm f/2 Macro 1:1 Di II Canon lens. The same manual settings, lighting, and height of the camera were used for all samples and the focusing was done manually. Microscopic pictures were made with a USB microscope (Dino‐Lite AM7013MZT4 and AM4515T8). There was no additional postprocessing or correction of images.

### 2.5 Metabolic activity

Metabolic activity of the samples was measured with a colorimetric assay using the tetrazolium salt MTT (Freimoser et al., 1999; Liu et al., 1997; Mosmann, 1983). This assay is based on the reduction of the yellow MTT to a purple colored formazan by mitochondrial enzymes (Riss et al., 2013). A filter paper sample was added to 1 ml of MTT solution (0.5 mg/ml) in a 12‐well plate. The plate was shaken for 16 ± 1 hr in an incubator shaker (150 rpm and 20°C–25°C). Subsequently, 2 ml of isopropanol were added and this was shaken for another 7 hr (150 rpm and 20°C–25°C). Then, the fluid was transferred to a centrifuge tube and centrifuged (15,000*g*, 5 min) to remove lysed cells and debris. The amount of formed formazan was recorded by measuring absorbance of the supernatant at 560 nm with a Shimadzu UV‐2600 spectrophotometer. Isopropanol was used as a blank.

To compare MTT absorbance of samples and controls measured at the same moment in time, a one‐tailed, unpaired Welch's *t* test with alpha 0.025 was used. A two‐tailed, unpaired Welch's *t* test with alpha 0.05 was used to compare MTT absorbance of different oils measured at the same moment in time. All statistical analyses were done in Microsoft Excel.

### 2.6 Hydrolysis of oil

**Figure 2:**
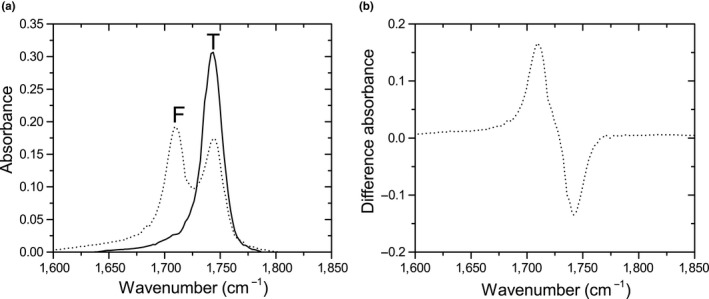
(a) Part of the FT‐IR spectrum of olive oil (solid line) and partly lipase‐converted olive oil (dotted line). The carbonyl bond (C=O) from the ester group in triglycerides shows an absorption band at ˜1744 cm^−1^ (T). The carbonyl bond in the carboxylic acid group of fatty acids absorbs at ˜1709 cm^−1^ (F). (b) The spectrum of partly lipase‐degraded oil subtracted by the spectrum of olive oil

The hydrolysis of oil by the fungus was studied with FT‐IR (Ismail et al., 1993). If the oil is hydrolyzed completely, each oil molecule (triacylglycerol) is converted into three free fatty acids and one glycerol molecule. Infrared absorbance of the samples was measured using a Shimadzu FTIR 8400S with a horizontal ATR (attenuated total reflectance) crystal. Before analysis, the crystal surface was thoroughly cleaned with a tissue soaked in isopropanol and left to dry. The filter sample was applied with the bottom toward the surface of the crystal and the spectrum was collected by coadding 24 scans at a resolution of 4.0 cm^−1^ and automatic gain. Part of the obtained spectrum from olive oil and partly lipase‐converted olive oil is shown in Figure 2a. The carbonyl bond (C=O) from the ester group in triglycerides absorbs infrared at ˜1,744 cm^−1^ (right peak, T). The carbonyl bond in the carboxylic acid group of fatty acids absorbs at ˜1,709 cm^−1^ (left peak, F) (Coates, 2000). The spectrum of pure oil was subtracted from the spectra (Figure 2b) and these differences in spectra were used for analysis.

To estimate oil conversion, the absorbance at 1,709 cm^−1^ of the corresponding blank oil (=*F*_blank_) was subtracted from the fatty acid peak of the sample (=*F*_sample_) and divided by the maximum value of the triglyceride peak of the corresponding blank oil at 1744 cm^−1^ (=*T*_sample_), as this is the maximum amount of carbonyl groups that can be converted (Equation (1)). It is assumed that the intensity of both peaks is equal, since the probed carbonyl groups are closely related. To compare conversion of the two oils a two‐tailed, unpaired Welch's *t* test with alpha 0.05 was used.

(1) $$\text{Oil conversion}=\frac{F_{\mathrm{sample}}-F_{\mathrm{blank}}}{T_{\mathrm{blank}}}$$

### 2.7 Lipase on oil

To compare hydrolysis of olive and linseed oil into glycerol and fatty acids by the enzyme lipase and to study water attraction by produced glycerol during this process, a commercially available lipase (from *Candida rugosa* via Sigma Aldrich) was applied on both oils. One Unit of lipase is defined as the amount of enzyme that hydrolyzes 1.0 microequivalent of fatty acid from olive oil in 1 hr at pH 7.2 at 37°C (incubation time 30 min). Lipase solutions (200 or 2,000 Units (U) per ml) were prepared in autoclaved demineralized water. A droplet of 5 μl of lipase solution containing 1 or 10 U lipase was applied on a hydrophilic PVDF filter with pore size 0.22 μm (Merck Millipore). The filter was dried at room conditions for at least 15 min until the fluid of the suspension has evaporated, and was placed on 20 μl of oil. The filters were incubated at the same conditions as the filters for the growth experiments, and at different time moments, FT‐IR spectra of two or three samples were obtained as described in the subsection hydrolysis of oil. To compare conversion of the two oils by lipase a two‐tailed, unpaired Welch's *t* test with alpha 0.05 was used. All statistical analyses were done in Microsoft Excel.

### 2.8 Mobility of oil followed by NMR

Nuclear magnetic resonance imaging was used to study changes in the mobility of the oil due to autoxidation and cross‐linking. A hydrophilic PVDF filter with pore size 0.2 μm (ø 13 mm, Merck Millipore) was placed on 20 μl of oil on a microscopic cover slip (18 × 18 mm, thickness 140 μm). The filters were incubated at the same conditions as the growth experiments. At certain moments in time, NMR measurements were performed using a GARField ‘H‐NMR setup with an electromagnet, generating a magnetic field of 1.4 T with a gradient of 42 T/m perpendicular to the filter sample (Glover et al., 1999). An Ostroff–Waugh pulse sequence was used to obtain the hydrogen density profiles and the signal decay: α_*x*_°−τ−[α_*y*_°−τ−echo−τ]_*n*_ (Ostroff & Waugh, 1966). The number of echoes (*n*) was 1,024. The interecho time (2τ) was 100 μs and the window width for recording the echo was 90 μs. Each profile consists of 2,048 averages with a repetition time of 1 s.

Every position in the sample shows a signal decay with a typical decay constant called *T*_2_. This *T*_2_ represents the spin–spin relaxation time (Slichter, 1996) and is dependent on the mobility of the hydrogen atoms. For instance, a hydrogen atom in liquid oil has a longer *T*_2_ than a less mobile hydrogen atom in cross‐linked oil. In order to determine the local mobility, signal decays at each position in the sample were fitted with an exponential decay function $\;S\left(t\right)=\;\sum_{i}A_{i}\text{exp}\left(\frac{-t_{e}*n}{T_{2,i}}\right)+\;S_{0}$. In this function, *A*_*i*_ is the amplitude, proportional to the hydrogen concentration, *t*_*e*_ is the echo time, *n* is the number of the echo, and *S*_0_ is the noise level. During autoxidation and cross‐linking of oil, triglyceride molecules form oligomers via an oxidative radical mechanism. Then, the layer of oil consists of a mixture of molecules with different mobility, having different *T*_2_ values. To study the distribution of the hydrogen groups with different *T*_2_ values, relative amplitudes were calculated by dividing each single amplitude (*A*_*i*_) by the total amplitude of all groups.

## 3 RESULTS

### 3.1 Growth on olive and linseed oil

Growth of *A. melanogenum* on linseed oil and olive oil was investigated. At days 3, 7, 16, 31, 64, and 106 of incubation, samples were imaged and analyzed with MTT assay and FT‐IR. These analyses are discussed in the following sections.

**Figure 3:**
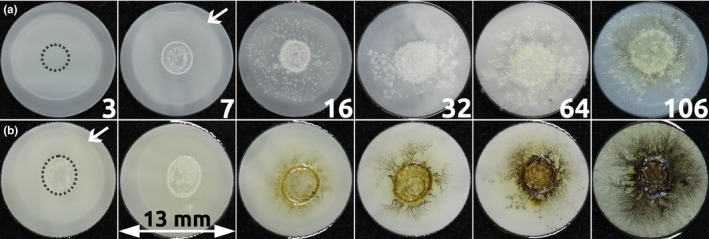
Overview pictures from *A. melanogenum* growing on a filter on olive oil (a) and linseed oil (b). Pictures are taken after 3, 7, 16, 31, 64, and 106 days of incubation. The diameter of the filter is 13 mm. The dashed circle marks the inoculation droplet. Arrows indicate the lighter circle due to water attraction

**Figure 4:**
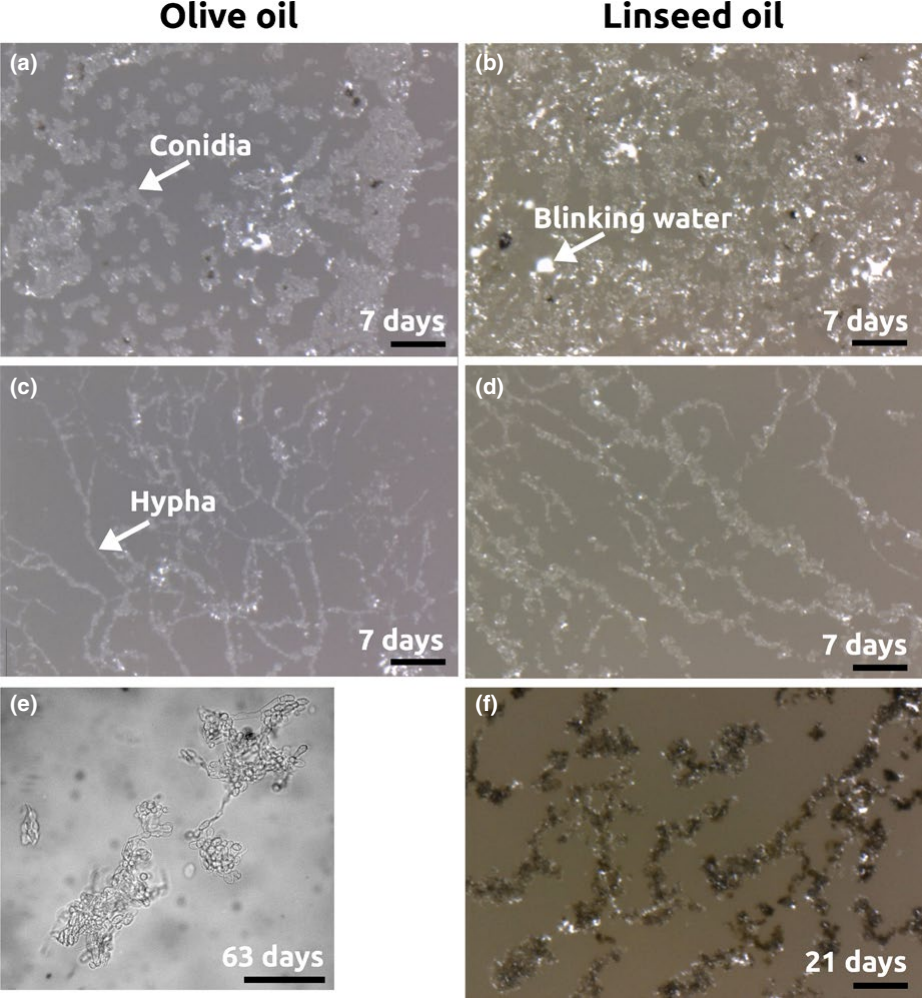
Microscopic pictures of *A. melanogenum* on a filter on olive oil (a, c, and e) and linseed oil (b, d, and f) after 7, 21, or 63 days of incubation. (a) and (b) inside inoculation droplet, (c) and (d) outside inoculation droplet, (e) hyphae‐like structures and conidia scraped from sample of olive oil, (f) Dark cells on linseed oil after 21 days of incubation. Scale bars: 100 μm (a–d and f) and 50 μm (e)

Figure 3a shows pictures of the filter samples with olive oil at days 3, 7, 16, 31, 64, and 106 of incubation. In the middle of the colony (i.e., the location of the inoculation droplet), growth was most dense and outside this region hyphae‐like structures are recognizable. Microscopic pictures (Figure 4) show that it is likely that yeast‐like cells or conidia are present, together with hyphae. Due to reflection of the samples, the exact structures are difficult to distinguish. Therefore, some material was scraped from the surface of the sample and studied using light microscopy (Figure 4e). This figure shows that indeed hyphae‐like structures together with conidia are present.

*Aureobasidium melanogenum* shows similar macroscopic (Figure 3) and microscopic (Figure 4a–d) structures on linseed and olive oil. Hyphae and conidia were observed on both oils. However, the color of the fungus is different at incubation times of 16 days and longer. Due to the formation of dark brown to black cells (Figure 4f), the colonies on linseed oil became brown between 7 and 16 days. In contrast to this, the colony on olive oil stayed white until day 64.

Water seems to be attracted by the samples, as can be seen on overview pictures, where the filter shows a circle with different (lighter) color (indicated by arrows in Figure 3). On microscopic pictures, the reflection is likely to be due to water. It is likely that this water is attracted by glycerol produced during oil hydrolysis. A similar effect was observed in the case of lipase applied on an oil‐saturated filter. It was observed that, in time, water was attracted by the samples, leading to a weight increase. The results of this experiment are discussed in a later section.

### 3.2 Metabolic activity

**Figure 5:**
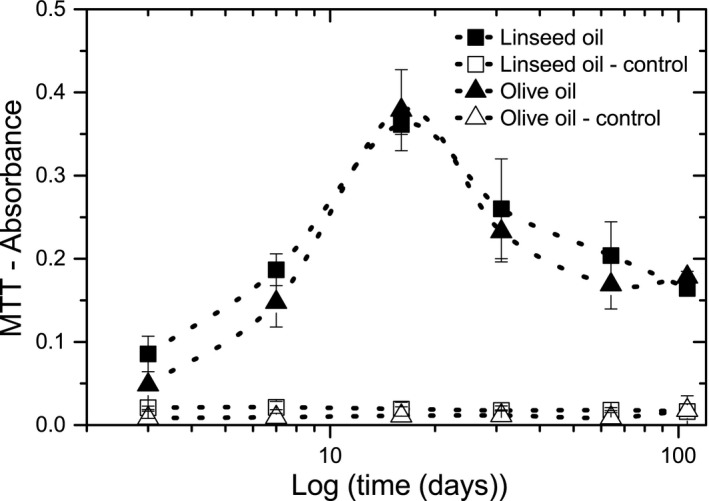
MTT absorbance (metabolic activity) of *A. melanogenum* on linseed and olive oil as a function of incubation time. Data points are averages of three samples, error bars represent the standard deviation. Control samples are without fungi

MTT analysis was used to compare metabolic activity of *A. melanogenum* on linseed and olive oil at several incubation times (Figure 5). MTT absorbance is linearly related to metabolic activity; the higher the absorbance, the more the metabolic activity. Metabolic activity on both oils shows the same trend; it increases until a maximum was measured at day 16, after which the activity decreases again. Moreover, on all analyzed time points no significant difference between metabolic activities on the two oils was observed.

### 3.3 Hydrolysis of oil

FT‐IR analysis was used to study the conversion of oil into fatty acids and glycerol. Control samples showed an identical spectrum with a single peak at ˜1,744 cm^−1^, which represents triglycerides (oil). Some minor fluctuations occur in the spectrum (±5%).

**Figure 6:**
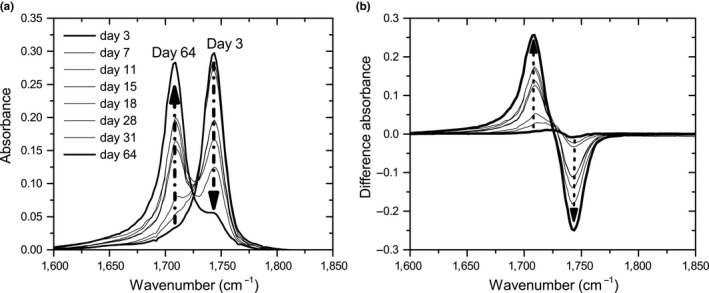
(a) FT‐IR spectra obtained from filters with *A. melanogenum* on olive oil (average of three samples). The spectrum of olive oil is subtracted and the difference spectra are plotted in Figure (b)

**Figure 7:**
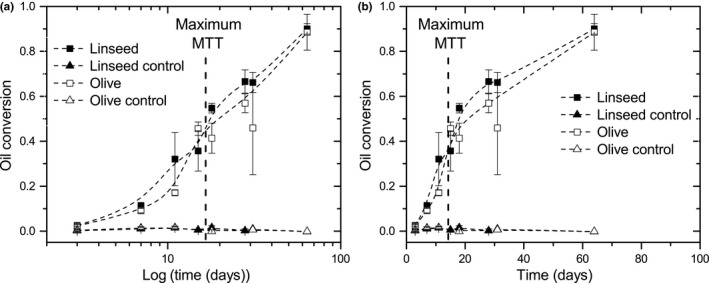
Conversion of linseed and olive oil into fatty acids by *A. melanogenum* as a function of incubation time (days). Data points are averages of three samples, error bars represent the standard deviation. Control samples are without fungi. (a) Oil conversion on a logarithmic timescale, (b) on linear timescale

The spectra from samples with *A. melanogenum* are shown in Figure 6a. The first spectrum obtained at day 3 shows a single peak around 1,744 cm^−1^. From day 7 onward, a second peak around 1,709 cm^−1^ becomes visible, which represents fatty acids. The fatty acid peak increases in time, whereas the triglyceride peak decreases simultaneously. This implies oil was converted into glycerol and fatty acids. The spectrum of olive oil was subtracted from all spectra, resulting in the difference spectra shown in Figure 6b. Oil conversion was calculated and is plotted in Figure 7 (open symbols).

The same analysis was done for linseed oil. The controls (triangles) stay at a constant low value, whereas samples of both olive and linseed oil show an increasing amount of fatty acids. This fatty acid formation continues at a lower rate after MTT has reached a maximum at day 16. On both oils, the same trend in fatty acid formation is observed. On all analyzed time points, except for day 7, no significant difference in oil conversion of both oils was observed. An average oil conversion of 0.885 and 0.900 for olive and linseed oil, respectively, was reached at day 64.

### 3.4 Mobility of oil followed by NMR

**Figure 8:**
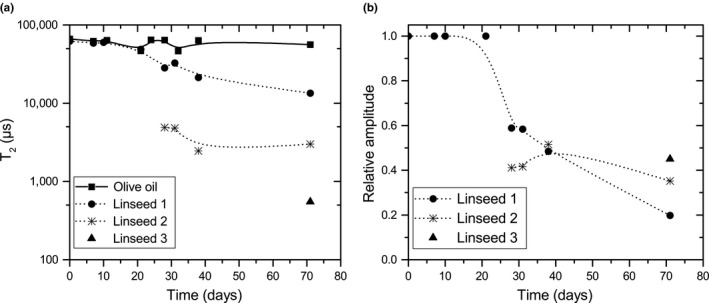
(a) *T*_2_ of filters with olive and linseed oil as a function of incubation time (days). (b) Relative amplitude (proportional to hydrogen concentration) of different hydrogen groups in linseed oil

To study changes in the mobility of the oil due to autoxidation and cross‐linking during the growth experiments, NMR was used. *T*_2_ values at several moments in time is shown in Figure 8a. The filter with olive oil (squares with continuous line) has a *T*_2_ of approximately 60 ms which was stable during the 0–71 days of incubation. The linseed oil sample (circles with dashed line) shows the same *T*_2_ as olive oil during the first 10 days and a decreased *T*_2_ at 21 days, meaning that the mobility of the hydrogen atoms is lower. After 28 days, two *T*_2_ values were observed belonging to two groups of hydrogen atoms with different mobility. Both *T*_2_ values decrease in time and after 71 days three different *T*_2_ values were observed.

To study the distribution of the different hydrogen groups with different *T*_2_ values observed in linseed oil, relative amplitudes were calculated and plotted in Figure 8b. The group of hydrogen atoms with highest *T*_2_ is decreasing in time. The groups with lower *T*_2_ values increase during the experiment. At 28, 31, and 38 days, the two groups of hydrogen atoms were present in approximately equal amounts. At 71 days, most hydrogen atoms in the sample belong to the group with lowest *T*_2_.

### 3.5 Lipase activity

**Figure 9:**
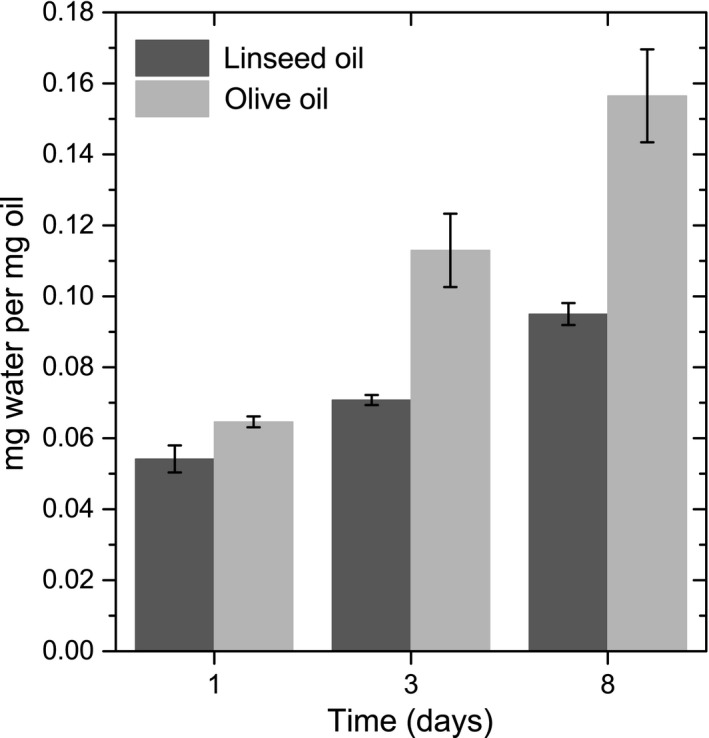
Water attraction due to oil conversion by 10 U lipase applied on a filter on oil. Data points are averages of three samples, error bars represent the standard deviation

*Aureobasidium melanogenum* is thought to consume oil by producing a lipase that converts oil into glycerol and fatty acids via hydrolysis. There was no significant difference in oil conversion found between olive and linseed oil in the fungal growth experiments. To test whether lipase has the same activity on olive and linseed oil, pure lipase (10 Units per filter) was applied to the oil. The amount of attracted water due to formation of glycerol in the process of oil hydrolysis was determined by drying and weighing of the samples. Figure 9 shows that in course of time water was attracted by the samples. Samples with olive oil attract more water than samples with linseed oil.

**Figure 10:**
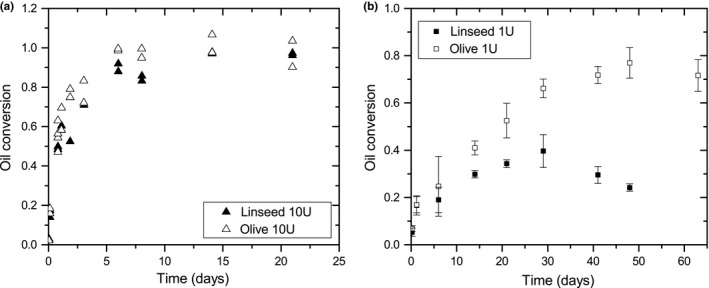
Oil conversion into fatty acids by lipase on a filter on linseed (closed symbols) and olive oil (open symbols). (a) 10 U (average of two samples) (b) 1 U lipase (average of three samples). Error bars represent the standard deviation in the oil conversion of the samples at a certain incubation time

To study lipase activity in more detail, the oil conversion was measured with FT‐IR. In Figure 10, oil conversion is plotted against time. Two different amounts of lipase (10 and 1 U per filter, Figure a and b, respectively) were applied on both oils, showing that the conversion rate depends on the amount of lipase. After 7 days, a maximum conversion close to 1.0 was reached for 10 U lipase. In this case, no significant differences between lipase activities on the two oils were observed. In case of 1 U lipase, after 14 days of incubation and longer, conversion of olive oil is significantly higher than linseed oil conversion. On olive oil with 1 U lipase, the maximum oil conversion was around 0.7 and was reached after approximately 50 days. The linseed oil samples that were incubated for 41 days and longer showed cross‐linking; the oil became more viscous and the infrared spectrum changed. Both triglyceride and fatty acid peaks decreased, which resulted in a lower value for oil conversion.

**Figure 11:**
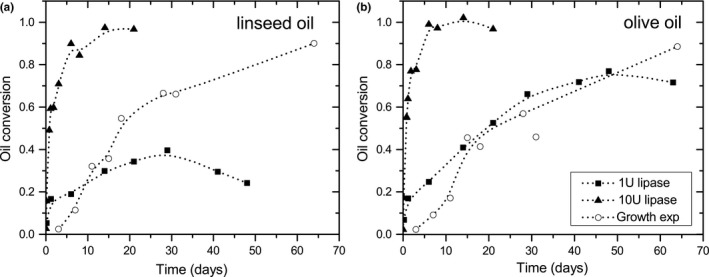
Linseed (a) and olive oil (b) conversion by lipase (1 U and 10 U, squares and triangles) and *A. melanogenum* (growth experiment, open circles)

In Figure 11, oil conversion by *A. melanogenum* is plotted together with the oil conversion by lipase. Oil conversion by lipase shows an exponential shape reaching a maximum oil conversion after approximately 7, 30, or 50 days depending on the oil type and the amount of lipase added. The maximum speed of the reaction was at the beginning. The curves from the growth experiments do not fit to this, but show a sigmoidal form until day 30 (circles). The maximum conversion rate was around 16 days, at the same time metabolic activity was also at maximum. In contrast to the lipase activity experiments, the oil conversion rate increases after a short plateau around 30 days. At the end of the growth experiments, oil was almost completely converted into fatty acids.

### 3.6 Growth on cross‐linked linseed oil

To study the effect of cross‐linking, linseed oil was heat‐treated until a rigid, cross‐linked layer was obtained and then inoculated with *A. melanogenum*. After 106 days of incubation, there was no visible growth observed. Also, no metabolic activity and no conversion of oil were detected. In addition, lipase activity test with 100 U lipase per filter did not show any conversion of oil.

## 4 DISCUSSION

In this study, growth of *A. melanogenum* on linseed and olive oil was investigated. Little is known about growth of *A. melanogenum* on vegetable oils as a single carbon source. Some other fungi, for instance, *C. rugosa* (Lakschmi et al., 1999; Serra et al., 1992) and several aspergilli (Reese et al., 1955) are known to be able to grow on linseed and olive oils. It is likely that *A. melanogenum* is able to use oil as nutrition, since it is known to produce lipase (Chi et al., 2009; Leathers et al., 2013; Leelaruji et al., 2013; Liu et al., 2008; Wang et al., 2007). It can grow on linseed oil‐based paints (Bardage, 1998; Harvath et al., 1976) and the oil is considered crucial for biofilm formation on oil‐impregnated wood (Van Nieuwenhuijzen et al., 2015). In this study, growth of *A. melanogenum* was observed on a filter on oil without any other carbon source available, confirming that *A. melanogenum* can use oil as a carbon source.

When the growth on olive oil and linseed oil were compared, *A. melanogenum* becomes dark sooner on linseed oil. This implies that linseed oil is stimulating melanin production and olive oil suppresses melanin production. Melanins are pigments that protect the fungus against environmental stresses, such as (ultraviolet) light, heat, oxidizing agents, and ionizing radiation (Bell & Wheeler, 1986; Eisenman & Casadevall, 2012; Jacobson, 2000). In contrast to olive oil, linseed oil is known to cross‐link, which leads to formation of radicals with free electrons. In such an environment, melanin can protect against these harmful free radicals (Gan et al., 1976; Jacobson, 2000; Mason et al., 1960). This may explain the earlier melanin production on linseed oil and the inhibitory effect of olive oil on melanin production.

Both macro‐ and microscopic observations showed apparent water attraction by the samples. It is assumed that water is attracted by glycerol. Glycerol is produced as a result of oil conversion and is hygroscopic. Water attraction due to glycerol was measured by applying lipase to oil and determining wet and dry weights as a function of time. It was observed that water is attracted by the samples to levels of 0.16 mg per mg oil. There was no fungus on these samples, so there are no other factors than glycerol production that can cause water attraction. The observed water attraction by the samples with *A. melanogenum* is therefore likely to be due to glycerol production. Together with the FT‐IR results, where fatty acid production is observed, it is confirmed that lipase is produced by *A. melanogenum* and is active outside the cell.

Metabolic activity and oil conversion by *A. melanogenum* were investigated. For samples with one cell type, MTT absorbance is linearly related to cell concentration. Our samples contain different cell structures (i.e., hyphae, yeast‐like cells, and/or chlamydospores) with unknown metabolic activity; therefore, it is not possible to calculate cell concentrations or biomass from the absorbance values and metabolic activity is expressed as MTT absorbance per filter sample. At the moment of maximum metabolic activity, the rate of fatty acid production is also at maximum confirming that MTT and FT‐IR address the correlated processes. The MTT assay measures the metabolic activity, which is needed to produce enzymes, while FT‐IR measures the product formed by the enzymes. This explains the shape of the curves from metabolic activity and oil conversion.

Oil conversion by *A. melanogenum* during growth experiments shows a different trend than oil conversion by lipase. The curves from lipase activity have an exponential shape reaching a maximum oil conversion after approximately 7 days for 10 U lipase on both oils, and 28–50 days for 1 U lipase for linseed oil and olive oil, respectively. The curves from the growth experiments do not fit to this, but have a sigmoidal form until day 30 which is typical for batch culture growth (Griffin, 1993). Initially, when the amount of biomass present is low, the fatty acid formation is low. Due to growth, the amount of lipase increases too, resulting in an acceleration of oil conversion. This is the reason why the rate of oil conversion increases around 10 days and was maximum at around 16 days, when metabolic activity was highest. In contrast with lipase experiments, oil conversion by the fungus increases and at the end of the growth experiments oil was almost completely converted into fatty acids and glycerol. During the growth of *A. melanogenum*, several processes occur. It can be speculated that lipase is not produced in equal amounts at every moment in time and fatty acids may be consumed by the fungus (Deacon, 2006), leading to a lower fatty acid peak and thus a lower value of oil conversion. All these processes lead to changes in the IR spectrum resulting in different oil conversion trends by the fungus and pure lipase.

During the first 14 days of incubation, no significant difference in oil conversion for 1 and 10 U lipase was observed. After that, olive oil conversion was significantly higher than linseed oil conversion by 1 U lipase. NMR experiments show a decreased *T*_2_ in linseed oil after 21 days of incubation, indicating that the oil is cross‐linking. Cross‐linking of oil starts with the formation of radicals (Porter et al., 1995), which is not visible with NMR. Therefore, it is likely that radicals are formed before 21 days of incubation. When applying 10 U of lipase, there was no difference in oil conversion of both oils. After 7 days, both oils were almost completely converted, before radical formation can play a role. From these observations, it can be speculated that formation of autoxidation products and cross‐linking of linseed oil diminish oil conversion by lipase.

In our growth experiments with *A. melanogenum*, no significant differences between conversion of linseed oil and olive oil were observed. As lipase conversion of olive oil was higher than conversion of linseed oil, it seems likely that *A. melanogenum* produces more lipase when grown on linseed oil instead of olive oil. This is in accordance with the literature that shows that lipase production by fungi is dependent on the carbon source in the medium (Lakschmi et al., 1999; Serra et al., 1992; Sun & Xu, 2008; Treichel et al., 2010). However, lipase produced by *A. melanogenum* can have a different activity than lipase from *C. rugosa*. Lipases from different species are known to show different hydrolysis activity toward certain carbon substrates (Song et al., 2008) and the position of a fatty acid on the glycerol backbone is important (Alford et al., 1964). Also, one species can produce more than one type of lipase (López et al., 2004). When assuming that lipase from *A. melanogenum* and *C. rugosa* are equally active toward the tested oils, it is rather strange that oil conversion goes to completion in the growth experiments where lipase experiments show an inhibitory effect of (cross‐linking of) linseed oil. Therefore, it can be hypothesized that growth of *A. melanogenum* prevents linseed oil from cross‐linking for instance by the production of slime or melanin (Jacobson, 2000). More research on lipase production by *A. melanogenum* and the effect of fungal growth on cross‐linking of linseed oil is necessary to confirm this.

Outdoor experiments showed an inhibitory effect of cross‐linked linseed oil on biofilm formation (Van Nieuwenhuijzen et al., 2015). In our study, no visible growth on severe cross‐linked linseed oil was observed, and also, neither metabolic activity nor degradation of the oil was detected. Furthermore, lipase activity experiments did not show any activity. Therefore, it can be concluded that cross‐linked linseed oil is not available for conversion into fatty acids and glycerol. Growth of *A. melanogenum* was suppressed dramatically.

To generate understanding of the growth mechanisms of the biofilm on oil‐impregnated wood, growth of *A. melanogenum* on different oil types was studied. In outdoor experiments on oil‐impregnated wood, the main observation regarding the influence of oil type on biofilm formation was that linseed oil led to biofilm formation only in combination with pine sapwood and olive oil stimulated biofilm formation regardless of the wood type (Van Nieuwenhuijzen et al., 2015). In contrast to this, our experiments do not show differences in metabolic activity and oil conversion of olive and linseed oil. Changes in the mobility of the oil were observed after 21 days of incubation. The growth of *A. melanogenum* was already observed before 21 days, and metabolic activity and oil conversion were at maximum rate around 16 days. In outdoor experiments, the growth was observed after cross‐linking of linseed oil occurs. Together with the growth experiments on severely cross‐linked linseed oil, where no growth was observed, this indicates that it is not the linseed oil itself, but process of cross‐linking of which slows down biofilm formation.

A different appearance of the colonies on linseed and olive oil was observed, although metabolic activity was equal. On linseed oil, the fungus produced more melanin than on olive oil. It is unknown how much energy is required for melanin production and how it effects the overall metabolic activity and growth of the fungus. A growth quantification method would gain more understanding of these processes. Several methods for fungal biomass quantification are well documented, for instance chitin (Fernandez & Koide, 2012; Vignon et al., 1986), glucosamine (Chysirichote et al., 2014), and ergosterol (Bjurman, 1994; De Ridder Duine et al., 2006). These methods are affected by the developmental stage and viability of the colony, sample preparation, and the presence of oil in the samples . For these reasons, it was very difficult to develop a reliable method.

### 4.1 Concluding remarks

From this study, it is concluded that *A. melanogenum* is able to grow on raw linseed oil and olive oil as a single carbon source. The fungus produces the enzyme lipase to convert the oil into fatty acids and glycerol. Metabolic activity was equal on linseed oil and olive oil. It was found that cross‐linking of linseed oil effects growth of *A. melanogenum*. The fungus was not able to grow on severely cross‐linked linseed oil, because the oil was not available for conversion into glycerol and fatty acids. This means that the degree of cross‐linking of the oil is important for growth of *A. melanogenum*. Colonies on linseed oil showed more melanin production than colonies on olive oil, probably due to formation of harmful free radicals during autoxidation and cross‐linking.

The results in this study show that lipase of *C. rugosa* converts more olive oil than linseed oil after a certain incubation time, probably due to inhibition by radical formation. Oil conversion by *A. melanogenum* does not show a difference between linseed and olive oil. Therefore, it is hypothesized that *A. melanogenum* prevents linseed oil from cross‐linking, possibly by the production of melanin.

The results in this study are not in accordance with the outdoor growth experiments on oil‐impregnated wood. Cross‐linking of linseed oil and the timescale on which growth starts seem to be important, but more research needs to be done to investigate this. To obtain more information about growth of *A. melanogenum* on oil, the development of a growth quantification method and studying cross‐linking of oil during growth are suggested for future work. In our experiments, oil conversion by *A. melanogenum* showed a maximum rate on both olive and linseed oil after which the rate decreases. A lipase activity measurement during growth would give more information about the exact mechanism of oil consumption by the fungus.

## CONFLICT OF INTEREST

The authors declare no conflict of interest.
